# FRAILTY AND DEPRESSIVE SYMPTOMS MEDIATE THE IMPACTS OF FOOD INSECURITY ON SLEEP DEFICIENCY AMONG OLDER FILIPINOS

**DOI:** 10.1093/geroni/igac059.3130

**Published:** 2022-12-20

**Authors:** Tuo-Yu Chen, Grace Cruz, Yasuhiko Saito

**Affiliations:** Taipei Medical University, Taipei City, Taipei, Taiwan (Republic of China); University of Philippines, Diliman, Quezon, Philippines; Nihon University, Chiyoda-ku, Tokyo, Japan

## Abstract

Although research has shown that food insecurity may lead to sleep problems due to mental illness, it remains unclear whether physical health also mediates this relationship in addition to poor mental health. We investigated whether frailty and depressive symptoms mediated the association of food insecurity with sleep deficiency (i.e., inadequate and poor sleep) among community-dwelling older adults. We analyzed the baseline data of the Longitudinal Study of Ageing and Health in the Philippines, a study with national representative sample of adults aged 60 years or older (Nf5,187). Sleep deficiency was conceptualized as self-reported sleeping less than 6 hours, complaining about trouble with falling asleep and staying asleep, and/or having non-restorative sleep. Food insecurity was defined as being hungry and not having enough food in the past three months in the household. Frailty was operationalized using modified definitions under Fried’s criteria. Depressive symptoms were assessed using the Center for Epidemiological Studies-Depression Scale. Covariates included socio-demographics (age,sex,education,wealth,insurance,living arrangement,urbanity), health (pain,chronic diseases), and lifestyles (smoking,drinking). Mediation analysis was performed using PROCESS macro. The results from bootstrapping showed that the indirect effects from food insecurity through frailty (b=.024,SE=.009,95%CI=.009-.043) and depressive symptoms (b =.118,SE=.022,95% CI=.078-.166) onto sleep deficiency were significant, but the direct effect from food insecurity to sleep deficiency was not (b=.243,SE=.134,95% CI=-.018-.505). The results suggested that the effects of food insecurity on sleep deficiency were fully mediated through frailty and depressive symptoms among community-dwelling older Filipinos. Preventing frailty and depression may help improve sleep health among individuals being food insecure.

